# Supplementary material for: Estimation of foveal avascular zone area from a B-scan OCT image using machine learning algorithms
Source: PLoS One. 2024 Dec 16;19(12):e0315825. doi: 10.1371/journal.pone.0315825 (PMC11649111; doi:10.1371/journal.pone.0315825)
Supplement: Attachment — Submitted filename: rebuttal_letter.docx [file pone.0315825.s004.docx]

Reviewer #1

Comment 1: Data Availability

Response:

Thank you for highlighting the importance of data availability. In response to your comment, we have uploaded all the OCT B-scan images, corresponding FAZ measurements, and the code used in our study to Figshare. The data are now publicly accessible at DOI: 10.6084/m9.figshare.27262257.v1. All data have been anonymized to protect patient privacy, adhering to the ethical guidelines approved by the Institutional Review Board of The University of Tokyo Hospital.

We have updated the Data and Code Availability section of the manuscript (page 12) to reflect this:

Data and Code Availability

The OCT B-scan images, corresponding FAZ measurements, and the code used in this study are publicly available on Figshare (DOI: 10.6084/m9.figshare.27262257.v1). All data have been anonymized to protect patient privacy, in accordance with the ethical guidelines approved by the Institutional Review Board of The University of Tokyo Hospital.

Comment 2: Overstated Conclusions

Response:

Thank you for your insightful feedback regarding the conclusions of our study. We agree that the original conclusions may have overstated the implications of our findings. In response, we have revised the Discussion section, including the concluding remarks, to more accurately reflect the scope of our results and to avoid overgeneralization.

Revisions made:

Discussion Section (pages 20-21):

We have revised the Discussion section to clarify the contexts in which our models might be applicable and to acknowledge the limitations of our study.

Clarified the applicability of our models:

Noted that while OCTA imaging is generally feasible, there are clinical situations where obtaining high-quality OCTA images may be challenging. This includes patients with severe cataracts, significant motion artifacts due to poor fixation, or limited cooperation often seen in pediatric or elderly populations.

Emphasized that our findings suggest the potential use of OCT B-scan images to estimate FAZ area in such situations.

Acknowledged limitations:

Expanded the existing "Limitations" subsection to explicitly discuss the limitations of our study, including:

The focus on healthy adult subjects without retinal vascular diseases, which may limit the generalizability of our models to other populations with retinal pathologies.

The potential limitations in applying our models to images obtained from different OCT devices or in suboptimal imaging conditions.

The need for further research to validate and potentially extend our models to broader populations, including patients with retinal diseases and different age groups.

By making these revisions, we aim to ensure that the discussion accurately reflects the scope and findings of our study without overstating our conclusions. We have tempered the language to align closely with our specific results, emphasizing the preliminary nature of our findings and the necessity for further validation.

We appreciate your guidance in helping us improve the clarity and focus of our manuscript.

Reviewer #2

Comment 1: Device Information

Response:

Thank you for bringing this to our attention. We have included the details of the OCT and OCTA devices used in our study in the Methods section.

Revisions made:

Programming Environment and Equipment Section (page 6):

Programming Environment and Equipment

In this study, B-scan OCT and en-face OCTA images were captured using the Cirrus 6000 (Carl Zeiss Meditec, Jena, Germany). Python version 3.8.10 was utilized as the programming language, and Google Collaboratory served as the development environment, with GPU acceleration employed to expedite machine learning computations.

Comment 2: Bland–Altman Analysis

Response:

We appreciate your valuable suggestion. We have conducted Bland–Altman analyses to evaluate the agreement between the predicted and actual FAZ area measurements for the estimates based on 1, 3, and 5 B-scan OCT images. The results have been incorporated into the manuscript.

Revisions made:

Abstract Section (page 2-3):

The results of the Bland-Altman analysis are summarized and reflected in the abstract. Specifically, we have included the following information.

Bland–Altman analysis showed improved agreement between predicted and actual FAZ areas with increasing B-scan images, evidenced by decreasing biases and narrower limits of agreement:

Methods Section (Statistical Analysis, page 12):

We added the Bland–Altman analysis procedure:

Statistical Analysis

Pearson's correlation coefficient was utilized to assess the correlation between predicted and true values of FAZ area. Bland–Altman analyses were conducted to evaluate the agreement between predicted and actual FAZ area measurements for each estimation method [20]. To compare the estimation error among the three Estimates, repeated measures ANOVA was conducted, followed by paired t-tests for between-group comparisons. As a correction for multiple comparisons, Bonferroni correction was applied. Estimation error was calculated using the absolute difference between the estimated values and the actual values. Statistical analysis was performed using Python version 3.8.10, with a significance level set at less than 0.05.

Results Section (Bland–Altman Analysis, pages 16):

We added a new subsection:

Bland–Altman Analysis

To further assess the agreement between the predicted and actual FAZ area measurements, Bland–Altman analyses were conducted for the estimates based on 1, 3, and 5 B-scan OCT images. The mean differences (biases), standard deviations (SDs), and 95% limits of agreement were calculated for each case.

For Estimate (1 line), the mean difference (bias) was -0.0367 mm², with a standard deviation of 0.0759 mm², resulting in 95% limits of agreement from -0.1854 mm² to 0.1120 mm² (Figure 4F).

For Estimate (3 lines), the mean difference was -0.0254 mm², with a standard deviation of 0.0605 mm², and 95% limits of agreement from -0.1440 mm² to 0.0932 mm² (Figure 4G).

For Estimate (5 lines), the mean difference was -0.0123 mm², with a standard deviation of 0.0452 mm², and 95% limits of agreement from -0.1009 mm² to 0.0763 mm² (Figure 4H).

These results indicate that as the number of B-scan OCT images increases, the agreement between the predicted and actual FAZ area measurements improves, as reflected by the decreasing biases and narrower limits of agreement.

Figures:

We have added the Bland–Altman plots as Figures 4F, 4G, and 4H, and updated the figure captions accordingly.

Discussion Section (page 19):

We have discussed the Bland–Altman analysis results:

The Bland–Altman analyses further supported these findings, showing decreased mean differences (biases) and narrower 95% limits of agreement as the number of B-scan images increased. Specifically, the bias improved from -0.0367 mm² for the 1-line estimate to -0.0123 mm² for the 5-line estimate, indicating reduced systematic error. The narrowing of the limits of agreement suggests enhanced consistency and reliability in the FAZ area predictions with more B-scan images (Figures 4F–H).

Reference Added:

[20] Bland JM, Altman DG. Statistical methods for assessing agreement between two methods of clinical measurement. Lancet. 1986;1(8476):307–310. doi:10.1016/S0140-6736(86)90837-8.

Comment 3: Use of SCP Slab

Response:

Thank you for this insightful comment. We acknowledge that measuring the FAZ in the full retinal slab can, in certain contexts, provide more comprehensive results and may offer increased accuracy, especially in pathological conditions. However, we chose to use the superficial capillary plexus (SCP) slab in our study for several important reasons:

Prevalence in Research and Clinical Practice:

The SCP FAZ measurement is widely utilized in numerous studies and is a common reference point in both clinical practice and research. As highlighted in multiple studies [12–14], the SCP FAZ has been extensively investigated and is considered a standard parameter for assessing retinal vascular health and disease progression.

Relevance to Specific Populations:

Our study aims to develop a method to estimate FAZ area in populations where OCTA imaging is challenging, such as children and the elderly. The SCP FAZ measurement has been particularly valuable in pediatric research. For instance, studies like the one by Lonngi et al. [15] have investigated SCP FAZ in children with amblyopia, demonstrating its applicability and importance in younger populations. Measuring the SCP FAZ aligns with our goal of facilitating FAZ assessment in these groups.

Consistency with Existing Literature:

Using the SCP slab allows for direct comparison with a large body of existing literature on healthy subjects and various disease states. This consistency enhances the applicability and interpretability of our findings within the broader context of retinal research.

Standard Clinical Protocols:

The SCP slab is routinely used in standard clinical protocols for FAZ assessment. Aligning our methodology with these protocols ensures that our results are relevant and easily translatable to everyday clinical settings.

While we recognize that measuring the FAZ in the full retinal slab can yield more comprehensive data, especially in certain pathological conditions, our focus was on establishing a foundational model using the most commonly utilized and clinically relevant approach. We believe that starting with the SCP slab provides a solid basis for future studies. We plan to explore FAZ measurements in the full retinal slab in subsequent research to assess whether our models can be adapted or extended to include these measurements, potentially improving accuracy and clinical applicability.

Changes to Manuscript:

In the "Image Acquisition and Preparation" subsection under Methods (page 7), we added:

Image Acquisition and Preparation

The FAZ area was measured using the superficial capillary plexus (SCP) slab of the en-face OCTA images. The SCP FAZ measurement is widely used in clinical practice and research and is associated with various retinal pathologies and developmental conditions [12–14]. Moreover, studies have utilized SCP FAZ measurements in pediatric populations, such as children with amblyopia [15], highlighting its significance in populations where OCTA imaging is challenging. While measuring the FAZ in the full retinal slab can yield more comprehensive results in some cases [16, 17], we selected the SCP slab to align with standard clinical protocols and facilitate comparison with existing literature. Future studies will explore the application of our models to full retinal slab measurements.

References Added:

[12] Su B, Zhu X, Yang K, et al. Age- and sex-related differences in the retinal capillary plexus in healthy Chinese adults. Eye Vis (Lond). 2022;9(1):38. doi:10.1186/s40662-022-00307-0. PMID: 36180939; PMCID: PMC9526290.

[13] Park JJ, Soetikno BT, Fawzi AA. Characterization of the middle capillary plexus using optical coherence tomography angiography in healthy and diabetic eyes. Retina. 2016;36(11):2039-50. doi:10.1097/IAE.0000000000001077. PMID: 27205895; PMCID: PMC5077697.

[14] Lavia C, Mecê P, Nassisi M, et al. Retinal Capillary Plexus Pattern and Density from Fovea to Periphery Measured in Healthy Eyes with Swept-Source Optical Coherence Tomography Angiography. Sci Rep. 2020;10(1):1474. doi:10.1038/s41598-020-58359-y. PMID: 32001769; PMCID: PMC6992636.

[15] Lonngi M, Velez FG, Tsui I, et al. Spectral-Domain Optical Coherence Tomographic Angiography in Children With Amblyopia. JAMA Ophthalmol. 2017;135(10):1086-91. doi:10.1001/jamaophthalmol.2017.3423. PMID: 28910439; PMCID: PMC5710487.

[16] Szpernal J, Gaffney M, Linderman RE, et al. Assessing the Sensitivity of OCT-A Retinal Vasculature Metrics. Transl Vis Sci Technol. 2023;12(8):2. doi:10.1167/tvst.12.8.2. PMID: 37531114; PMCID: PMC10405864.

[17] Waheed NK, Rosen RB, Jia Y, et al. Optical coherence tomography angiography in diabetic retinopathy. Prog Retin Eye Res. 2023;97:101206. doi:10.1016/j.preteyeres.2023.101206. PMID: 37499857; PMCID: PMC11268430.

By providing this detailed explanation and updating the manuscript accordingly, we hope to have addressed your concern regarding our choice of the SCP slab for FAZ measurement. Thank you again for your valuable feedback, which has helped us improve the clarity and context of our methodology.

Comment 4: Inclusion of Only Healthy Subjects

Response:

Thank you for highlighting this important limitation. We agree that including only healthy adult subjects without retinal vascular diseases may restrict the applicability of our machine learning models to populations with retinal pathologies.

Revisions made:

Discussion Section (Limitations, page 20):

Limitations

A significant limitation of our study is the inclusion of only healthy adult subjects without retinal vascular diseases. This focus may restrict the applicability of our machine learning models to populations with retinal pathologies. Previous research has demonstrated that in diseased eyes, even the built-in software of OCTA devices can be inaccurate in delineating the FAZ area [23]. Therefore, our models, trained on data from healthy eyes, may not generalize well to eyes with retinal diseases.

We have cited the reference you suggested to support this point.

Reference Added:

[23] Mirshahi R, Anvari P, Riazi-Esfahani H, et al. Foveal avascular zone segmentation in optical coherence tomography angiography images using a deep learning approach. Sci Rep. 2021;11(1):1031. doi:10.1038/s41598-020-80058-x. PMID: 33441825; PMCID: PMC7806603.

Additional Revisions

In addition to the changes made in response to the reviewers' comments, we have addressed the following:

Data Availability

The OCT B-scan images, corresponding FAZ measurements, used in this study are publicly available on Figshare (DOI: 10.6084/m9.figshare.27262257.v1).

Funding Statement:

We have clarified the role of the funders in the study.

Funding Statement

This work received funding from the Japan Society for the Promotion of Science (JSPS) KAKENHI (Grant Number: 22K16963) specifically to cover the publication fees. The funders had no role in study design, data collection and analysis, decision to publish, or preparation of the manuscript.

Supporting Information Captions:

We have included captions for our Supporting Information files at the end of the manuscript and updated the in-text citations.

Supporting Information

S1 Code. Python code for Model 1 used to predict FAZ length from B-scan OCT images.

S2 Code. Python code for Model 2 used to predict FAZ area from FAZ length measurements.

S3 Code. Python code for Model 3 used to convert FAZ area from pixels to mm².

Reference List Verification:

We have reviewed the reference list to ensure that it is complete and accurate, following the journal's guidelines.

We hope that these revisions comprehensively address your comments and enhance the quality and clarity of our manuscript. We are grateful for your thoughtful feedback, which has helped us improve our work significantly.

Please let us know if there are any further concerns or suggestions.

We are committed to further improving our manuscript and are happy to address any additional comments or concerns you may have. Thank you once again for your valuable feedback.

Sincerely,

Taku Toyama
